# The effects of lobbying on the FDA’s recall classification

**DOI:** 10.1186/s12910-023-00921-0

**Published:** 2023-06-20

**Authors:** Yifan Zhou

**Affiliations:** Fanhai International School of Finance, Fudan University, 220 Handan Road, Yangpu District, Shanghai, 200433 China

**Keywords:** Recall, FDA, Conflict of interest, Lobbying

## Abstract

**Background:**

The US Food and Drug Administration (FDA) regulates goods accounting for 20% of US consumers’ total expenditure. The agency’s potential susceptibility to corporate lobbying and political influence may adversely affect the its abilities to fulfill its duties as a vital federal agency. This study assesses whether the FDA’s product recall classifications in recall scenarios are influenced by firms’ lobbying activities.

**Methods:**

The universe of all FDA recalls between 2012 and 2019 is obtained from the FDA’s website. Firm names are matched to federal-level lobbying data obtained from the Center for Responsive Politics – a non-profit and nonpartisan organization that tracks lobbying expenditures and campaign contributions. Analyses are conducted using ordinary-least-squares regressions, in which the dependent variable is recall classification and independent variables are three different measures of firms’ lobbying activities in the one year prior to the recall.

**Results:**

Firms that engage in lobbying appear more likely to receive favourable classifications from the FDA. When examining the above results by product type, we find that classification of food recalls seems to be subject to lobbying influence, but the same does not appear to be true for drug and device recalls. Evidence is consistent with the conjecture that the distinction between medical and food firms may be a result of medical firms targeting lobbying efforts at FDA approvals, rather than recalls.

**Conclusions:**

Between 2012 and 2019, the FDA’s product recall classifications seem to be significantly influenced by firms’ lobbying activities. Lobbying firms appear to have received more favorable (i.e., less severe) recall classifications compared to non-lobbying firms.

## Background

The US Food and Drug Administration (FDA) regulates $2.6 trillion of goods, including food, medical products, and tobacco. These products account for approximately 20 percent of all expenditure by US consumers [1]. One key function of the FDA is its continuous monitoring of products under its regulation for safety and quality. Should a product be deemed hazardous, the FDA has the statutory authority to mandate recalls. Recalls are actions taken by a firm, often the manufacturer, to remove the product from the market.

The Center Recall Unit (CRU) within the FDA is responsible for recalls. In each recall scenario, the FDA would first conduct or obtain health hazard evaluations (HHEs). Based on the report, the CRU determines the appropriate recall strategy, considering factors such as precedent HHEs of products that are similar to the one in question. The CRU then classifies the recall, before setting audit strategies and any recommendations.

FDA recalls are commonplace. In 2019, there were 1,570 product recalls, of which 720 were for drugs, 396 were for medical devices, and 454 were for food. All FDA recalls are classified into one of three classes, depending on the severity of the health hazard [2].

- Class I – a situation in which there is a reasonable probability that the use of, or exposure to, a violative product will cause serious adverse health consequences or death.
- Class II – a situation in which use of, or exposure to, a violative product may cause temporary or medically reversible adverse health consequences or where the probability of serious adverse health consequences is remote.
- Class III – a situation in which use of, or exposure to, a violative product is not likely to cause adverse health consequences.

The FDA’s recall classification guidelines require and allow for subjective judgments to be made when determining a recall’s class. This paper examines whether firms’ past lobbying activities, whether targeted at the FDA or more broadly at the federal level, have any influence on the FDA’s recall classification decisions.

Lobbying in the US typically operates through professional lobbying firms (that sometime double as law, public relations, or accounting firms) hired by clients (e.g., pharmaceutical companies) [3]. The Lobbying Disclosure Act of 1995 mandates that lobbying firms provide a good-faith estimate, rounded to the nearest $10,000, of all lobbying-related income from their clients [4]. Companies with in-house lobbyists are required to do the same – provide a good-faith estimate, rounded to the nearest $10,000, of all lobbying-related expenditure [4]. Lobbying reports are filed with the Secretary of the Senate or the Clerk of the House of Representatives on a quarterly basis [4]. Filing is exempt for those that spend less than $3,000 on lobbying in the quarter [4].

Pharmaceutical companies actively contribute to election campaigns and engage in lobbying activities [5–8]. Between 1999 and 2018, the pharmaceutical and health product industry spent $4.7 billion (inflation adjusted) on lobbying at the federal level, more than any other industry [8]. Although companies do not disclose the dollar amount spent lobbying each agency or politician, it is not unreasonable to assume that a significant fraction of this total lobbying expenditure is, directly or indirectly, targeted at the FDA, given the agency’s power and authority over products under its regulation.

Previous studies have examined this issue from various perspectives. In 2016, a study published in *The BMJ*found that over 57 percent of FDA medical reviewers worked for or consulted to biopharmaceutical companies after leaving the FDA [9]. Similarly, an examination of 107 physicians who advised the FDA between 2008 and 2014 by the *Science*magazine found that 62 percent of those physicians received money for travel or consulting, or received research subsidies from either the makers of drugs on which they previously reviewed or those makers’ competing firms [10].

There is anecdotal evidence to suggest that FDA decisions could be prone to influence and pressure. For example, in 2008, the FDA approved a knee implant – Menaflex [11]. The green light came following political pressure from congressmen whom received campaign contributions from ReGen Biologics – maker of Menaflex [11]. Two years later, the FDA stated that it “should not have approved” the device in the first place, as “the Menaflex device is intended to be used for different purposes and is technologically dissimilar from devices already on the market” and subsequently rescinded the implant’s clearance over safety concerns [12]. ReGen Biologics responded to the FDA’s decision to rescind Menaflex’s 510(k) clearance by stating that there is “absolutely no substance to the FDA’s assertion that ReGen used undue political influence to secure Menaflex’s 510(k) review or its clearance.” [13].

Even those within the FDA recognize the agency’s political vulnerability. In 2018, a bipartisan group of seven former FDA Commissioners advocated for making the FDA an independent agency by breaking it out of the Department of Health and Human Services, with the aim of making the FDA less susceptible to political pressure [14].

## Methods

### Data

The FDA’s recall data is publicly available from the FDA’s website. Data was obtained for all FDA recalls between 2012 and 2019. Three types of products could be recalled by the FDA – food, drugs, and medical devices. Each recall is classified into one of three classes based the health risks that the situation imposes, as determined by the FDA.

Lobbying data is obtained from the OpenSecrets – a non-profit and nonpartisan organization that is dedicated to “tracking money in politics”, including federal campaign contributions and lobbying data. I download all companies that lobbied the FDA between 2011 and 2019, including the number of reports filed and the total dollar amount spent lobbying at the federal level (not necessarily targeted at the FDA, as firms no do have to disclose how lobbying expenditure is apportioned) each year. From the FDA’s recall dataset, I extract names of recall firms and match those with names of lobbying clients.

### Statistics

This paper examines the research question using multivariate ordinary-least-squares (OLS) regression analyses. Huber-White standard errors are used to allow for heteroscedastic residuals. Since recalls from the same firm may be related (e.g., similar quality control standards), standard errors are also clustered at the firm level. Stata 15 is used for all statistical analyses presented in this paper.

I adopt an OLS regression framework:

1 $${Class}_{i,j,t}={\beta }_{1}{X}_{j,t-1}+{\gamma }_{t}+{\varepsilon }_{i,j,t}$$

where *i*, *j*, and *t* index recall, firm, and year, respectively. *Class* take the value of one, two, or three – corresponding to the FDA’s recall classification. *X* is one of three measures of lobbying – (i) *Lobby* is a dummy that equals to one if the firm lobbied the FDA in the one year prior to the recall and zero otherwise, (ii) *Reports* measures the number of lobbying reports filed by the firm (or by its lobbyist on behalf of the firm) in the one year prior to the recall (one may view this as a proxy for the firm’s lobbying activeness – filing every quarter likely indicates more active lobbying compared to filing every second quarter), and (iii) the dollar amount, in millions, spent on lobbying in the one year prior to the recall. The inclusion of year dummies (${\gamma }_{t}$) absorbs any unobservable fixed effects across recalls in any given year. One may also view *Class* as an ordinal variable, in which case ordered logistic regressions should be used instead of OLS regressions. Untabulated results show that findings are unchanged when using ordered logistics regressions.

## Results

There was a total of 46,522 recalls between 2012 and 2019, of which 40,330 recalls were related to non-lobbying firms and 6,192 recalls were related to lobbying firms (Table 1). Lobbying firms are associated with more recalls per firm compared to non-lobbying firms. The average non-lobbying firm is responsible for 7.81 recalls (40,300 / 5,158 = 7.81) and the average lobbying firm is responsible for 32.94 recalls (6,192 / 188 = 32.94). Lobbying firms’ recalls are also conditionally less severe, with a mean class of 1.82 compared to non-lobbying firms’ mean class of 2.00 (Table 1).

**Table 1:** Descriptive statistics by recall product type

		Non-Lobbying Firms (*N* = 5,158)	Lobbying Firms (*N* = 188)
***All Recalls***	*N*	40,300	6,192
Class	1 (%)	22.01	5.49
	2 (%)	72.28	89.02
	3 (%)	5.72	5.49
	Mean (SD)	1.82 (0.52)	2.00 (0.30)
Report Number	Mean (SD)	0 (-)	0.41 (1.14)
Dollar Amount ($M)	Mean (SD)	0 (-)	0.20 (1.11)
***Food Recalls***	*N*	17,436	217
Class	1 (%)	43.71	0
	2 (%)	50.56	100
	3 (%)	5.73	0
	Mean (SD)	1.61 (0.59)	1.88 (0.37)
Report Number	Mean (SD)	0 (-)	0.01 (0.07)
Dollar Amount ($M)	Mean (SD)	0 (-)	0.01 (0.16)
***Drug Recalls***	*N*	8,955	899
Class	1 (%)	11.61	5.59
	2 (%)	77.86	82.17
	3 (%)	10.52	12.24
	Mean (SD)	1.98 (0.46)	2.09 (0.56)
Report Number	Mean (SD)	0 (-)	0.91 (1.47)
Dollar Amount ($M)	Mean (SD)	0 (-)	0.89 (2.60)
***Device Recalls***	*N*	13,939	5,076
Class	1 (%)	6.63	5.45
	2 (%)	90.16	92.44
	3 (%)	3.21	2.11
	Mean (SD)	1.96 (0.34)	1.98 (0.22)
Report Number	Mean (SD)	0 (-)	0.34 (1.06)
Dollar Amount ($M)	Mean (SD)	0 (-)	0.09 (0.44)

**Fig. 1:**
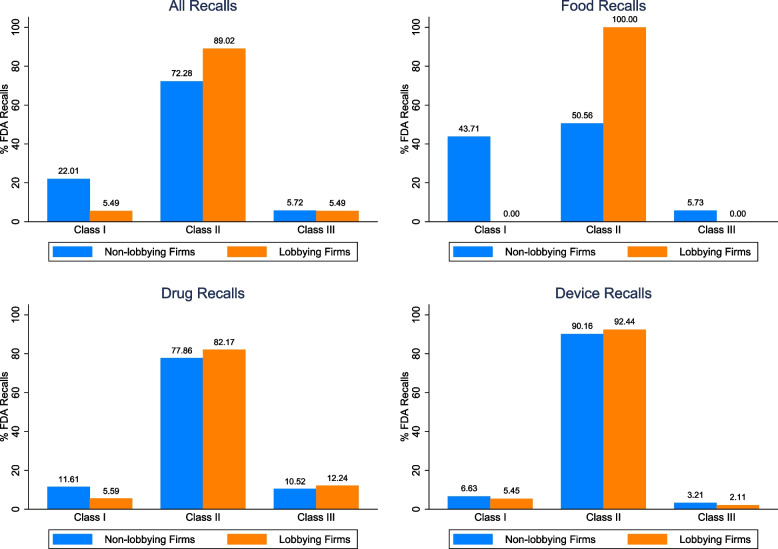
FDA recall distribution across lobbying and non-lobbying firms between 2012 and 2019

The notion that lobbying firms are more likely to receive more favorable recall classifications is supported by the fraction of recalls within each class for lobbying and non-lobbying firms (Fig. 1 top-left panel). The blue (orange) bars represent lobbying (non-lobbying) firms’ recalls. Between 2012 and 2019, lobbying firms were more likely to receive Class II classification compared to non-lobbying firms. This is offset by lobbying firms’ lower likelihood of receiving Class I classification compared to non-lobbying firms. Similar patterns are observed for each product type.

We see that all three measures of lobbying lead to statistically significant results and provide evidence consistent with my hypothesis (Table 2 Panel A). Column 1 suggests that recalls of firms that lobbied the FDA in the previous year are 0.145 (95% CI: [0.052, 0.239]) classes higher. Column 2 suggests that each additional lobbying report filed lead to an increase in class of 0.045 (95% CI: [0.016, 0.073]). Columns 3 suggests that recall class increases by 0.309 (95% CI: [0.041, 0.577]) per million dollar increase in lobbying expenditure at the federal level.

**Table 2:** Pooled OLS regressions showing that lobbying activities lead to less severe FDA recall classification between 2012 and 2019. Huber-White robust standard errors are clustered by firm; 95% confidence intervals are shown in square brackets; *p*-values are shown in parentheses; ∗  ∗  ∗ and ** indicates statistical significance at the 1% and 5% levels, respectively

*Dependent variable:*	Class		
	(1)	(2)	(3)
***Panel A: All Recalls***			
Lobby	0.145***		
	[0.052, 0.239]		
	(0.002)		
Reports		0.045***	
		[0.016, 0.073]	
		(0.002)	
Amount ($M)			0.309**
			[0.041, 0.577]
			(0.024)
N	46,519	46,519	46,519
Adjusted R-squared	0.024	0.024	0.024
***Panel B: Food Recalls***			
Lobby	0.567***		
	[0.426, 0.708]		
	(< 0.001)		
Reports		0.567***	
		[0.426, 0.708]	
		(< 0.001)	
Amount ($M)			1.012***
			[0.760, 1.264]
			(< 0.001)
N	17,652	17,652	17,652
Adjusted R-squared	0.043	0.043	0.043
***Panel C: Drug Recalls***			
Lobby	0.022		
	[-0.128, 0.172]		
	(0.771)		
Reports		0.021	
		[-0.026, 0.068]	
		(0.385)	
Amount ($M)			0.112
			[-0.342, 0.566]
			(0.629)
N	9852	9852	9852
Adjusted R-squared	0.135	0.136	0.136
***Panel D: Device Recalls***			
Lobby	-0.004		
	[-0.089, 0.082]		
	(0.935)		
Reports		-0.002	
		[-0.024, 0.020]	
		(0.867)	
Amount ($M)			-0.048
			[-0.245, 0.150]
			(0.637)
N	19,015	19,015	19,015
Adjusted R-squared	0.035	0.035	0.035

Having established that lobbying appears to lead to more favorable recall classifications, we move on to examine whether this phenomenon is present across all products types. Panels B, C, and D of Table 2 restricts recalls related to food, drugs, and devices, respectively. Interestingly, we see that results in Panel A are driven almost exclusively by food recalls. In other words, it appears that only food manufacturers’ lobbying efforts influence the FDA’s recall classifications; pharmaceutical and medical device companies’ lobbying efforts do not.

## Discussion

In the previous section, we noted that FDA recall classifications appear to be influenced by firms’ lobbying activities in the one year prior to the recall. More specifically, lobbying firms are seemingly more likely to receive less severe recall classifications compared to non-lobbying firms. One interesting aspect of this finding is that it only appears in food recalls, but not in drug and device recalls. This is perhaps unsurprising given the common belief that pharmaceutical companies are far more concerned with drug approval than perhaps any other FDA action. Consistent with this belief, we observe that 82 percent of lobbying firms’ recalls are medical product-related, whereas this figure is only 57 percent for non-lobbying firms’ recalls (Table 1). This indicates that medical firms may be more likely to lobby the FDA compared to food firms, possibly suggesting their greater reliance on the FDA’s actions, such as approval decisions. Indeed, previous research has found that lobbying reduces the number of days to approval [15]. The fact that food recalls may be susceptible to political influence is concerning. The nature of food products means that they likely reach and impact a larger fraction of the population compared to drugs and devices. By extension, this also implies that they may pose a greater danger to public health. Media and public attention tend to focus on “big pharmas”, but food companies should not be neglected.

Another interesting aspect of the results is that our coefficient of interest across all three columns in Table 2 loses statistical significance once firm dummies are included in the model. This likely suggests that both the identity of lobbying firms and their lobbying expenditure do not change much over the years. In other words, lobbying firms persistently lobby, and spend similar amounts on lobbying, over our sample period. This is consistent with the notion of companies exerting political influence to achieve their objectives. In fact, lobbying expenditures of firms such as Pfizer, Gilead, Amgen, and Merck are consistently ranked in the top ten within the pharmaceutical industry [16]. In 2022, other familiar names such as Johnson & Johnson, Eli Lilly, Bristol-Myers Squibb, AstraZeneca, and Bayer are also featured in the top twenty [16]. The fact that companies with large lobbying outlays are regularly receiving favorable treatments, both in recall and approval scenarios [15], could be concerning.

Congress has a number of weapons under its disposal to address the concerns around the FDA’s susceptibility to political pressure. First, the FDA currently falls under the jurisdiction of the Department of Health and Human Services (HHS) – a cabinet-level executive branch department, and therefore partisan. Breaking the FDA out of the HHS and making it an independent agency would reduce its susceptibility to political pressure. Second, Congress could introduce reforms on revolving door – personnel movement between capacity as legislators/regulators and industry members. Third, Congress could further reform lobbying disclosure requirements. Although a number of laws aimed at making lobbying more transparent have been introduced since the 1990s (e.g., Lobbying Disclosure Act of 1995, Lobbying Transparency and Accountability Act of 2006, and Honest Leadership and Open Government Act of 2007), the lobbying industry remains shrouded in mystery. Insiders acknowledge that the exact size of the lobbying industry is difficult to estimate, by at least twice as much is spent that officially reported [17]. One would like to hope that Congress would act swiftly given the overwhelming evidence suggesting the problematic actions of companies under FDA jurisdiction. However, given that these firms contribute significantly to politicians’ campaigns [8], the situation seems bleak.

### Limitations

Analyses conducted in this paper are not without limitations. First, filing requirements dictate that firms that spend less than $3,000 in the quarter do not have to report their lobbying activities. This means that the lobbying dataset is missing firms who engage in lobbying on a smaller scale. If small-scale lobbying also translates to less severe recall classifications, then misclassifying small-scale lobbying firms as non-lobbying firms would have led to an underestimation of coefficient magnitude. In other words, our current estimation of lobbying’s effect on recall classifications is understated. As such, our current regression results are conservative estimates of the true effect of lobbying on recall classification.

Second, lobbying activities may be somewhat correlated to firms’ financial observables (e.g., total revenue). Thus, it would be ideal to incorporate firms’ financials into multivariate regression analyses as potential confounders. However, due to the desire to study the entire universe of FDA recalls between 2012 and 2019, the paper incorporate all firms, both publicly listed and privately held. The nature of privately held firms means that they do not disclose their financial statements, and therefore making the inclusion of their financial observables impossible.

Third, the finding that lobbying apparently leads to less severe recall classifications could be interpreted differently due to confounding factors. For instance, firms that actively engage in lobbying may also have superior quality products compared to their otherwise similar competitors. In this case, the finding of this paper would not be unexpected, nor would it call into question the impartiality of the FDA. Given that this study chooses not to gauge product quality given a lack of objective measures across different product categories, it is worth noting this alternative explanation of the findings.

Fourth, data used in this study’s analyses is between 2012 and 2019. Subsequent recalls by the FDA are not considered. It may be the case that the findings of this paper no longer stand when the latest recall data is considered. Finally, whilst any commentary from the FDA may provide insights, the agency does not appear to have issued any public statements regarding its susceptibility to lobbying efforts.

## Conclusions

Firms’ lobbying activities, whether targeted at the FDA or more generally at the federal level, appears to have significant influence over the FDA’s recall classification. Firms that engage in lobbying appear more likely to receive favorable recall classifications from the FDA. Evidence is also consistent with the notion that compared to recall classifications, FDA approval decisions are of greater importance to pharmaceutical companies and medical device manufacturers.

It is perhaps worth noting that the purpose of this paper is not to solely focus the FDA’s recall practice, but rather using recalls to reflect a potentially greater concern regarding the impartiality of the FDA – a federal agency with significant responsibility and influence over every citizen’s daily life.

## Data Availability

The datasets used and/or analysed during the current study are available from the corresponding author on reasonable request.

## Abbreviations

CRU: Center Recall Unit
HHE: Health hazard evaluations
HHS: Department of Health and Human Services
FDA: Food and Drug Administration
OLS: Ordinary least squares
